# EIF3D promotes gallbladder cancer development by stabilizing GRK2 kinase and activating PI3K-AKT signaling pathway

**DOI:** 10.1038/cddis.2017.263

**Published:** 2017-06-08

**Authors:** Fei Zhang, Shanshan Xiang, Yang Cao, Maolan Li, Qiang Ma, Haibin Liang, Huaifeng Li, Yuanyuan Ye, Yijian Zhang, Lin Jiang, Yunping Hu, Jian Zhou, Xuefeng Wang, Yong Zhang, Lei Nie, Xiao Liang, Wei Gong, Yingbin Liu

**Affiliations:** 1 Department of General Surgery, Xinhua Hospital Affiliated to Shanghai Jiao Tong University School of Medicine, Shanghai, China; 2 Shanghai Research Center of Biliary Tract Disease, Shanghai, China; 3 Department of Molecular and Cellular Oncology, The University of Texas MD Anderson Cancer Center, Houston, TX, USA; 4 Department of General Surgery, Sir Runrun Shaw Hospital Affiliated to Zhejiang University, Hangzhou, China

## Abstract

Recent evidence suggests that dysregulated eIF3d expression may be critical in various genetic disorders as well as cancer. In this study, we observed that EIF3d levels increased in gallbladder cancer (GBC) samples compared with non-tumor tissue. High eIF3d levels were associated with advanced tumor stage and metastasis and were correlated with poor prognosis in 92 patients with GBC. Depletion of EIF3d in GBC cell lines inhibited cell proliferation, colony formation and metastasis and induced apoptosis and cell cycle arrest *in vitro* and *in vivo*. In contrast, ectopic expression of eIF3d had the opposite effects. Moreover, in this study, we revealed that a novel non-translational factor function of eIF3d mediated its protumoral effects. In details, eIF3d stabilizes GRK2 protein by blocking ubiquitin-mediated degradation, consequently activates PI3K/Akt signaling, and promotes GBC cell proliferation and migration. In conclusion, eIF3d promotes GBC progression mainly via eIF3d–GRK2–AKT axis and it may be used as a prognostic factor. The therapeutic targeting of eIF3d–GRK2 axis may be a potential treatment approach for GBC.

Gallbladder cancer (GBC) is the most common biliary tract malignancy and the seventh most common gastrointestinal cancer with an incidence of 2.5 per 100 000 persons according to the SEER program.^1, 2, 3^ Despite the relatively low incidence rate, owing to its nonspecific symptoms and highly invasive nature, most patients are at an advanced stage when they are diagnosed. The median survival time for individuals with GBC is <1 year.^4^ Most of these patients die from tumor metastasis and recurrence. Therefore, an understanding of the molecular mechanisms of GBC metastasis and recurrence is indispensable for the development of effective adjuvant therapy.

Deregulated protein synthesis and degradation contribute to cancer genesis and progression. Control of protein synthesis directs both global and selective translation of specific mRNAs, the protein products in turn promote tumor cell survival, angiogenesis, invasion and metastasis.^5^ Protein synthesis in eukaryotes is primarily regulated at initiation, the rate-limiting step that involves a large number of eukaryotic initiation factors (eIFs). EIF3 is the largest (650 kDa) subunit of the most complex initiation factors. Mammalian eIF3 is composed of at least 13 individual subunits designated eIF3a-eIF3m^6^ and dysregulation of eIF3 subunit expression correlates to cancer development and progression.^7^ EIF3a has been shown to be aberrantly expressed in several human cancers, including lung, breast, colon, esophagus and cervical cancers, and its depletion by antisense cDNA reversed the malignant phenotype in human cancer.^8, 9, 10, 11, 12^ EIF3b has been reported to be overexpressed in colon cancer cells and related to human bladder and prostate cancer prognosis^13, 14^ Overexpression of a truncated eIF3e mutant caused malignant transformation of mammary epithelial cells both *in vitro and in vivo*.^15^ EIF3h is frequently amplified in breast, prostate cancer and non-small cell lung cancer alone with the adjacent MYC pro-oncogene and its overexpression promotes cancer cell growth.^16, 17^ Recently, eIF3d is continually investigated in cancers. Sudo *et al.*^18^ first identified eIF3d as one of genes involved in mesothelioma cell viability using a genome-wide small interfering RNA library. EIF3d was also associated with malignant transformation of aberrant karyotypic human embryonic stem cells.^19^ Most recently, a study shows eIF3d is required for specialized translation initiation, regulating the target protein translation.^20^

However, these data fall short of implicating eIF3d as a prognostic factor and molecular mechanism requirement for the maintenance or progression of human cancer. Here we show that eIF3d is upregulated in GBC and strongly associated with poor patient outcome. EIF3d promotes GBC cell proliferation, migration and tumor growth *in vitro and in vivo*. Moreover, we revealed a novel non-translational factor function of eIF3d, which inhibits specialized protein degradation. EIF3d physically interacts with the GRK2 and, as a result, abrogates the ubiquitination and degradation of GRK2 to promote GBC progression. Taken together, our results reveal a novel mechanism by which eIF3d control the target protein stability via posttranslational mechanism.

## Result

### EIF3D is aberrantly expressed in GBC and correlates with poor survival in GBC patients

**Figure 1:**
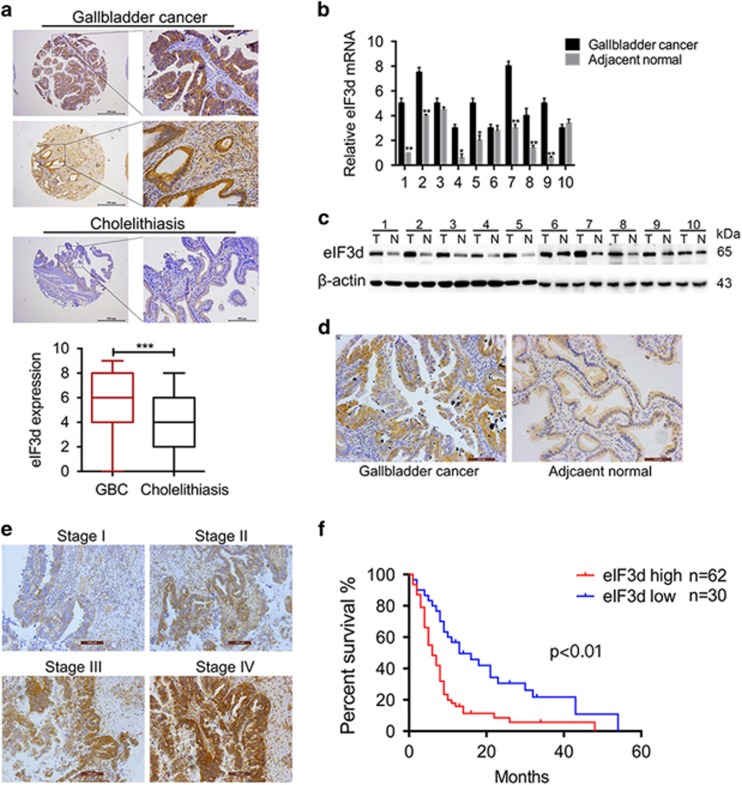
EIF3d is overexpressed in GBCs and correlated to GBC progression and poor survival of patients. (**a**) Upper panel, representative images of immunohistochemical staining of EIF3D protein expression levels in GBC (92 tumor samples) and non-tumor (cholecystitis, 103 cases) tissues. Bottom panel, quantification of EIF3D protein expression in GBC samples compared with normal tissues. (***P<0.001, Mann–Whitney *t*-test). (**b**-**d**) EIF3D protein expression levels in representative primary GBC tissues (T) and their paired non-tumor tissues (N) was evaluated by qPCR (**b**), western blotting (**c**) and IHC analyses (**d**) (*P<0.05, **P<0.01). (**e**) Representative IHC staining images of GBC patient samples with clinical stages I, II, III and IV, respectively. (**d**) Kaplan–Meier curve of the overall survival of GBC patients based on EIF3D expression. EIF3D ^high^, *n*=62; EIF3D ^low^, *n*=30

Recently, some studies have reported that eIF3d was involved in the development and progression of several types cancer cells.^21, 22^ To explore whether eIF3d expression level is associated with pathological features and disease progress, we determined eIF3d expression levels in the gallbladder tumor and non-tumor tissues by IHC staining. The expression of eIF3d levels was increased significantly in 92 human tumor samples as compared with the 103 cholecystitis gallbladder epithelial tissues (Figure 1a). Furthermore, the mRNA and protein expression levels of eIF3d were measured in 10 pairs of human GBC specimens and their matched normal tissues by qRT-PCR, western blot and IHC. The expression level of eIF3d was significantly higher in tumor tissues than that in their non-tumor counterparts (Figures 1b and d). To further investigate the correlation of eIF3d with GBC progress, we analyze the eIF3d expression in 92 GBCs. A clinicopathologic association study in GBCs demonstrated that overexpression of eIF3d was significantly associated with advanced clinical stage (Figure 1e), and lymph node and liver metastasis (Table 1). These data imply that eIF3d may have an important role in GBC progression and metastasis. The Kaplan–Meier analysis on overall survival *versus* eIF3d expression in 92 patients was shown in Figure 1f. The prognosis for patients with low eIF3d expression was significantly better than that for patients with higher eIF3d expression (*P*<0.01).

### EIF3d promotes GBC cell growth

**Figure 2:**
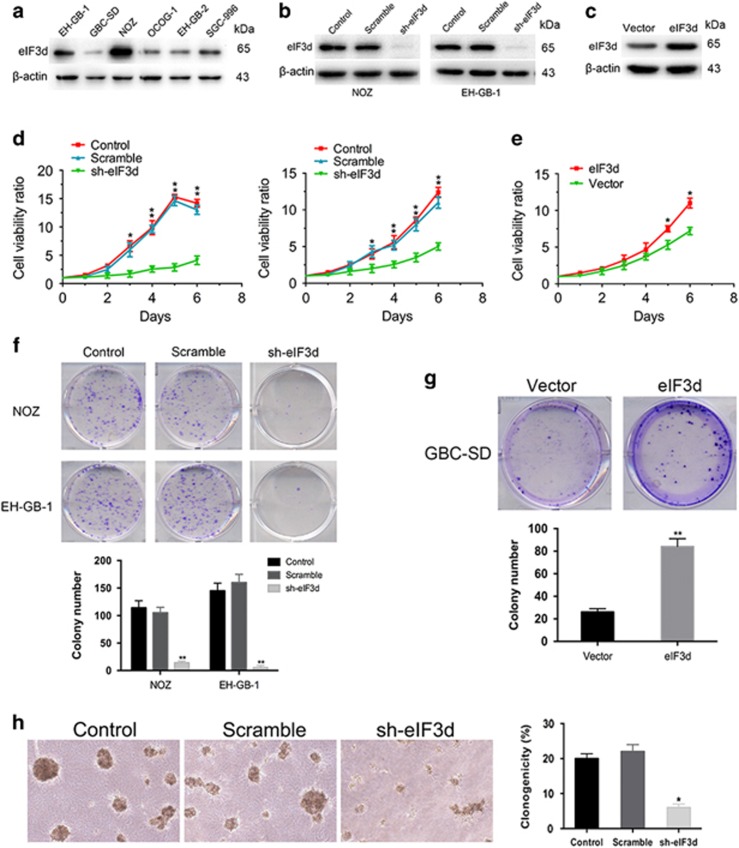
EIF3d promotes GBC cell growth. (**a**) The expression levels of eIF3d protein in six human GBC cell lines. Whole-cell lysates were immunoblotted with specific antibodies against eIF3d protein. (**b**) Lentivirus-mediated eIF3d knockdown in NOZ and EH-GB-1 cells. (**c**) Ectopic expression of eIF3d in GBC-SD cell lines. (**d**) Cells proliferation was inhibited in eIF3d knocked down NOZ and EH-GH-1 cells. (**e**) Overexpression of eIF3d promoted cell growth in GBC-SD cell. (**f**) Knockdown of eIF3d suppressed colony formation in NOZ and EH-GH-1 cells. (**g**) Overexpression of eIF3d promoted colony formation in GBC-SD cells. (**h**) Colony formation in soft agar assay of eIF3d knockdown and control NOZ cells. Error bars indicate the mean±S.E. of three independent experiments. (*P<0.05, **P<0.01)

To explore the role of eIF3d in the progression of GBC, we first detected the endogenous expression of eIF3d in six GBC cell lines. EIF3d was highly expressed in NOZ and EH-GB-1 cells but weakly expressed in GBC-SD cell (Figure 2a). Interestingly, our previous study shows NOZ and EH-GB-1 cells are highly proliferative cancer cells, whereas GBC-SD cells are slowly proliferative cancer cells.^23^ These data suggest eIF3d may contribute to regulating GBC cell proliferation. To test the hypothesis, we knocked down with shRNA against eIF3d in NOZ and EH-GB-1, whereas ectopic overexpressed eIF3d in GBC-SD cell. The efficiency of knockdown and overexpression of eIF3d were evaluated by qPCR and western blot (Figures 2b, c and Supplementary Figure 1) and we chose the best one shRNA (shRNA#2) for the following study. Meanwhile, we found silencing of eIF3d did not significantly affect the expression levels of the core subunits of eIF3 in GBC cells (Supplementary Figure 2).

Next, we examined the biological effects under both eIF3d knockdown and overexpression conditions in GBC cells. As shown in Figure 2d, cell proliferation was significantly suppressed by eIF3d knockdown in both NOZ and EH-GB-1 cells. In contrast, the cell proliferation was significantly enhanced by overexpression of eIF3d in GBC-SD cells (Figure 2e). In addition, eIF3d knockdown attenuated the colony formation capability of NOZ and EH-GB-1 cells (Figure 2f), whereas eIF3d overexpression in GBC-SD cells promoted colony formation as compared with vector control cells (Figure 2g). Furthermore, knockdown of eIF3d decreased the colony formation ability in soft agar assay (Figure 2h), suggesting eIF3d may be required for an anchorage-independent growth of GBCs.

### Silencing of eIF3d leads to G0/1 cell cycle arrest and promotes apoptosis in GBC cells

**Figure 3:**
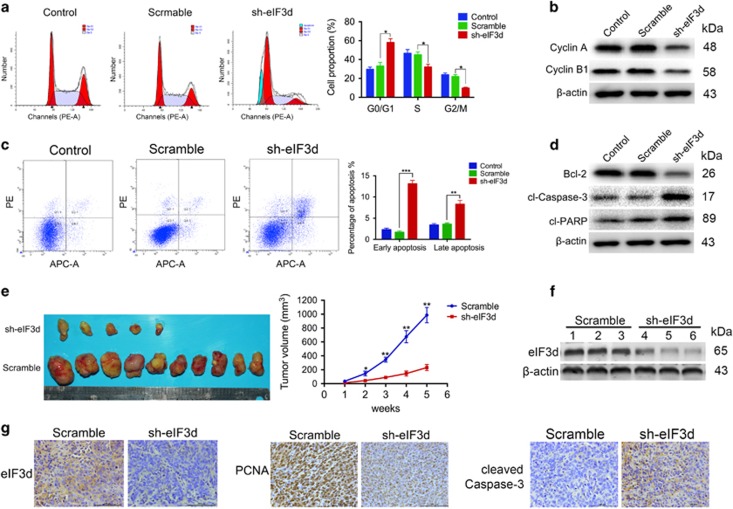
Silencing of eIF3d leads to G0/1 cell cycle arrest and promotes apoptosis in GBC cells. (**a**) Flow cytometry analysis of the cell cycle of eIF3d knockdown and scramble control cells. Error bars indicate the mean±S.E. of three independent experiments (*P<0.05). (**b**) The cell cycle regulator phospho-p21 (p-P21) and cyclin B1 in whole-cell lysates were detected by western blot analysis with indicated antibodies, *β*-actin was used as loading control. (**c**) Cell apoptosis was detected by flow cytometry with annexin V/PI staining. Error bars indicate the mean±S.E. of three independent experiments (**P<0.01, ***P<0.001). (**d**) Total cell lysates were immunoblotted with antibodies against Bcl-2, cleaved caspase-3 and cleaved-PARP. (**e**) Tumor sizes were measured every week. The mean and S.D. were calculated from at least five independent samples. *P<0.05, **P<0.01 *versus* control. (**f**) Protein expression of eIF3d in GBC xenografts. (**g**) The expression of eIF3d, PCNA and cleaved caspase-3 was detected in GBC xenografts by IHC

To determine the mechanism by which eIF3d promoted cell proliferation, we analyzed the effect of eIF3d on cell cycle by flow cytometry (Figure 3a). The results showed that eIF3d knockdown in the NOZ cells led to a significant reduction in the S and G2/M phase population, but increases G1 phase population. Consistently, eIF3d knockdown reduced the expression levels of cyclin A and cyclin B1 (Figure 3b). These findings indicate that eIF3d acts on the G1-S and S-G2/M checkpoint to promote the cell cycle in GBC cells. These results showed that depletion of eIF3d induced cell cycle arrest. In addition, in the eIF3d shRNA group, an increased cell proportion was observed in the sub-G1 population (Figure 3a). These prompted us to further examine the contribution of apoptosis to the observed growth inhibition in NOZ cells by eIF3d knockdown. Flow cytometry analyses with annexin V and PI showed an increase in the number of early apoptotic cells and the late apoptotic cells in the eIF3d knockdown cells compared with the scramble cells (Figure 3c). We examined the key cell apoptosis regulators and found that eIF3d knockdown significantly reduced the protein levels of Bcl-2 and increased the cleaved caspase-3 and poly ADP ribose polymerase (PARP) protein levels (Figure 3d). These data suggested EIF3D is involved in regulation of GBC cell cycle and apoptosis.

To verify the positive role of eIF3d in gallbladder tumor progression *in vivo*, we performed xenograft tumor assays using eIF3d knockdown stable NOZ cells. We found that eIF3d knockdown significantly inhibited xenograft tumor formation and growth in nude mice (Figure 3e). We evaluated the knockdown efficiency of eIF3d in transplanted tumor tissues from the mice by immunoblotting and IHC staining (Figures 3f and g). IHC staining with specific antibody against PCNA indicated in the eIF3d depletion tumor sections that there were fewer proliferative cells, however, increased staining intensity for cleaved caspase-3 was observed, indicating significantly increased apoptosis cells in eIF3d shRNA xenograft tumors (Figure 3g). Collectively, these data support our hypothesis that eIF3d acted as a novel tumor-promoting molecule and positively regulates gallbladder tumor growth.

### EIF3d promotes GBC cells migration *in vitro* and *in vivo*

**Figure 4:**
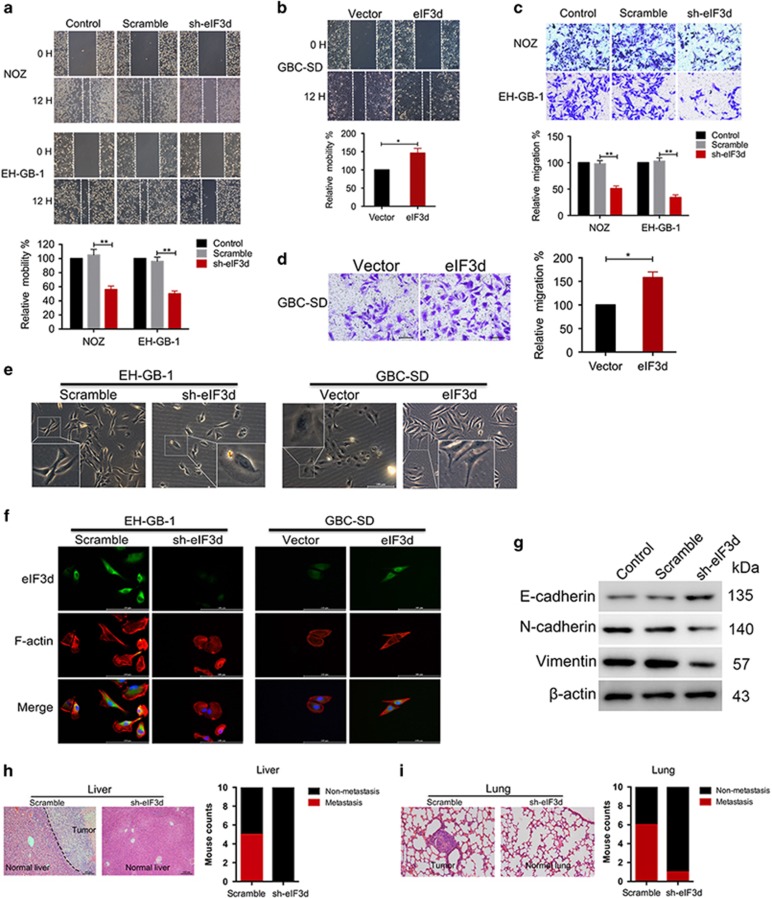
EIF3d promotes cell migration and invasion *in vitro* and *in vivo* by inducing EMT. (**a**) Decrease of migration of NOZ (left) and EH-GB-1 (right) cells with eIF3d knockdown in wound-healing assay. Relative migration rate was shown in the bar graph. (**b**) Ectopic expression of eIF3d in GBC cells increased migration. Relative migration was shown in the bar graph. (**c**) Transwell migration assays of NOZ and EH-GB-1 cells expressing eIF3d and scramble shRNAs. The bar graphs show the relative migration potential after 12-h culture. (**d**) Transwell migration assays of GBC-SD cells expressing eIF3d and control cells. The graphs represent the migration rates of the cancer cells after culture on the transwell for 12 h. Error bars indicate the mean±S.E. of three independent experiments (*P<0.05, **P<0.01. (**e**) Morphologies of EH-GB-1 and GBC-SD cells were examined under a microscope. (**f**) Confocal images of EH-GB-1 (left) and GBC-SD (right) cells transfected with eIF3d shRNA and eIF3d expression vector stained for eIF3d (green), F-actin (red), and DAPI (blue). (**g**) Effect of eIF3d knockdown on epithelial and mesenchymal markers. Total cellular proteins were immunoblotted with the indicated specific antibodies against E-cadherin, N-cadherin and Vimentin, respectively. (**h**) Depletion of eIF3d by shRNA knockdown reduced liver metastasis. The nude mice were transplanted with 2 × 10^6^ of EH-GB-1 cells (scramble and sh-eIF3d) in the spleen. Representative photos of histological H&E staining of liver metastasis were shown for each group. A bar graph summarizes the incidence of liver metastasis in the two groups. (**i**) Knockdown of eIF3d by shRNA reduced lung metastasis. The nude mice were injected with 2 × 10^6^ of EH-GB-1 cells (scramble and sh-eIF3d) via the lateral tail vein. Representative photos of histological H&E staining of lung metastasis were shown for each group. A bar graph summarizes the incidence of lung metastasis in the two groups

The clinicopathologic association study in GBCs revealed that overexpression of eIF3d was associated significantly with lymph node metastasis and liver metastasis (Table 1). These imply that eIF3d may have a role in metastasis. To investigate the effects of eIF3d on GBC cancer cell migration, the monolayer wound-healing assay and Boyden Transwell chamber assays were performed. Knockdown eIF3d markedly suppressed the cell mobility and migration ability by 50–70% in the NOZ cells and EH-GB-1 cells (Figures 4a and c), whereas forced expression of eIF3d had reversal effect on GBC-SD cells (Figures 4b and d). In addition, eIF3d markedly affected GBC cells invasive ability (Supplementary Figure 3). These data were consistent with our finding that increased eIF3d is significantly associated with advanced clinical stage and metastasis. The morphology of EH-GB-1 showed shuttle shape or multiple angle shape, and the multiple apophysis showed irregular shape. However, EH-GB-1 cells with eIF3d knockdown inhibited cell pseudopodia formation and became oval shape, whereas overexpression eIF3d in GBC-SD cell resulted in EMT morphological changes (Figure 4e). Immunofluorescence staining showed the eIF3d and cytoskeletal proteins F-actin expression in GBC cells (Figure 4f). Interestingly, compared with EH-GB-1 scramble control cells, the morphology of eIF3d knockdown cells lacked thin and long pseudopods. In contrast, overexpression of eIF3d in GBC-SD cell had the opposite effect. Moreover, the protein expression of the epithelial–mesenchymal transition (EMT) markers, including E-cadherin, N-cadherin and Vimentin, in the eIF3d knockdown cells was examined by western blot. Knockdown eIF3d enhanced the protein levels of E-cadherin, whereas reduced N-cadherin and Vimentin in the NOZ cells (Figure 4g). In order to assess the contribution of eIF3d to tumor metastasis *in vivo*, we used the animal model of experimental liver metastasis and pulmonary metastasis. We inoculated the eIF3d shRNA cell and scramble cells through spleen and tail intravenous injection in immunodeficient nude mice. Tumor nodules in the liver and lung carried higher incidence in those mice inoculated with scramble cells compared with those mice injected with the eIF3d knockdown cells, as revealed by gross examination and H&E staining (Figures 4h and i). These data indicated that eIF3d were critical for the migration of GBC cells by modulating the key EMT regulating factors.

### EIF3d directly interacts with GRK2 and enhances the protein stability of GRK2 by blocking the ubiquitin-mediated proteasome degradation of GRK2

**Figure 5:**
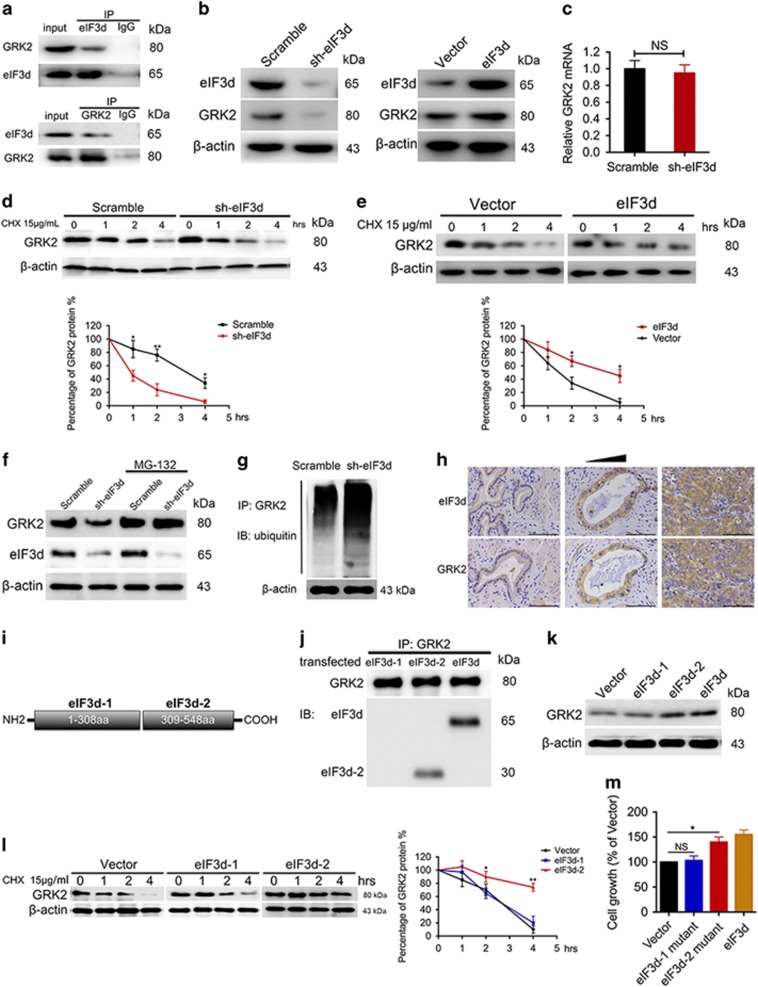
EIF3d interacts with and enhances the protein stability of GRK2 by reducing the ubiquitin–proteasome-mediated degradation. (**a**) EIF3d physically interacts with GRK2. The endogenous proteins from NOZ cells were immunoprecipitated with IgG or antibodies against eIF3d and GRK2, followed by western blot analysis and 10 % lysis for input. (**b**) Knockdown or ectopic expression of eIF3d affects GRK protein levels. Immunoblotting assay was performed to detect the GRK2 protein levels in the GBC cell lines expressing eIF3d or knocking-down eIF3d. (**c**) Real-time PCR detected the GRK2 mRNA expression in the eIF3d knockdown and control NOZ cell. (**d**) EIF3d expression level regulates stability of GRK2 protein. Knockdown of eIF3d was performed in NOZ cells, followed by the treatment with cycloheximide (CHX, 15 mg/ml) for various times as indicated. Whole-cell lysates were then analyzed by immunoblotting with the indicated antibodies (upper). The relative GRK2 protein level was determined using densitometry scanning (NIH ImageJ software, Bethesda, MD, USA) (down). (**e**) Ectopic expression of eIF3d increases GRK2 stability. EIF3d expression vector or empty vector was transfected into in GBC-SD cells. Treatment of cycloheximide and immunoblotting were performed as described in **d**. Error bars indicate the mean±S.E. of three independent experiments (*P<0.05, **P<0.01). (**f**) The regulation of GRK2 stability by eIF3d is through proteasome-mediated ubiquitination and degradation pathway. The NOZ cells were transfected with eIF3d shRNA or scrambled shRNA. At 48 h post transfection, the cells were treated with either vehicle or MG132 (1 mg/ml) for an additional 4 h. The whole-cell lysates were subjected to the immunoblotting with the indicated antibodies. (**g**) Depletion of eIF3d increases ubiquitination of GRK2. The scramble and eIF3d shRNA–infected cell lysates were immunoprecipitated with anti-GRK2 antibodies. The immunoprecipitates was then analyzed by immunoblotting with anti-ubiquitin (UB) or GRK2 antibodies. (**h**) EIF3d protein level was positively correlated to GRK2 protein level in different human GBC tissues. The representative IHC images were shown from human gallbladder primary tumor tissues. (**i**) Schematic representation of cDNA constructs for each eIF3d deletion mutant. (**j**) Cell lysates were obtained from GBC-SD cells transiently transfected with eIF3d and eIF3d deletion mutants. The resultant lysates were immunoprecipitated and immunoblotted. (**k**) Immunoblotting assay was performed to detect the GRK2 protein levels in the GBC cell lines transfected with eIF3d and eIF3d deletion mutants. (**l**) EIF3d truncation vector or empty vector was transfected into in GBC-SD cells. Treatment of cycloheximide and immunoblotting were performed as described in **d**. (**m**) GBC-SD cell transfected with empty vector, eIF3d and eIF3d mutant expression vector was cultured for 5 days. CCK-8 assay detected the cell viability

To further define the molecular basis for tumor-promoting function mediated by eIF3d, we performed the yeast two-hybrid assay with eIF3d as bait and have identified six eIF3d-interacting candidates (data not shown). Among those candidates, GRK2 has shown high significance of interaction with eIF3d. To validate the functional interaction between eIF3d and GRK2 in the cells, reciprocal co-immunoprecipitation assay was performed (Figure 5a). As expected, a positive GRK2 signal was observed in the co-immunoprecipitates pulled down by anti-eIF3d-specific antibody. Moreover, eIF3d was also detected in the protein pool pulled down by anti-GRK2 antibody. These results confirmed there is physical interaction between eIF3d and GRK2 in GBC cell line. In the light of the interaction between eIF3d and GRK2, we tested whether ectopic expression of eIF3d could alter the expression of GRK2. We determined the GRK2 expression level in the eIF3d knocked down cells, and found that depletion of eIF3d led to a decrease of GRK2 protein level. In the other hand, ectopic expression of eIF3d expression in GBC-SD cells led to an increase of GRK2 protein level (Figure 5b). These data indicated that eIF3d had positive regulatory effect on GRK2 expression. The elevated protein levels could be due to either enhanced mRNA expression or protein level regulation. Our observation showed that eIF3d knockdown did not affect GRK2 mRNA levels (Figure 5c). However, ablation of eIF3d with shRNA enhanced the degradation of GRK2 protein, compared with scramble shRNA in NOZ cells (Figure 5d). Conversely, transfection of the eIF3d expression vector in GBC-SD cells delayed the degradation of GRK2 protein (Figure 5e). Moreover, eIF3d knockdown-mediated enhancement in GRK2 protein degradation was abrogated by the treatment with proteasome inhibitor, MG132 (Figure 5f). These findings suggest that ubiquitin–proteasome system is involved in the degradation of GRK2. To further confirm this hypothesis, we examined ubiquitinated GRK2 in the eIF3d knockdown cells, as polyubiquitination is a signature of protein being degraded via ubiquitin-mediated proteasome system. Consistently, we found that knockdown of eIF3d enhanced the polyubiquitination of the endogenous GRK2 proteins in NOZ cells (Figure 5g). Furthermore, the expression patterns of eIF3d and GRK2 exhibited strong concordance in sections of human GBC tissues (Figure 5h). EIF3d expression significantly correlated with GRK2 expression in 92 human GBC specimens (Spearman correlation coefficient R=0.550; *P*<0.01) (Table 2) and 6 GBC cell lines (Spearman correlation coefficient R=1; *P*<0.01) (Supplementary Figure 4). To map the region of eIF3d necessary for the interaction and stabilization function with GRK2, we constructed truncation vectors of eIF3d according to the strucure and sequence alignment (Figure 5i).^20^ As shown in Figure 5j, GRK2 was co-precipitated with eIF3d and eIF3d-2 deletion mutants but not with the eIF3d-1 deletion mutant. Moreover, ectopic expression of eIF3d and eIF3d-2 but not eIF3d-1 expression in GBC-SD cells could upregulate GRK2 protein level (Figure 5k). In addition, transfection of the eIF3d-2 truncation vector but not eIF3d-1 truncation vector in GBC-SD cells delayed the degradation of GRK2 protein and promoted cell proliferation compared with transfection of empty vector (Figures 5l and m). Taken together, these results suggest that eIF3d especially the C-terminal region of eIF3d can increase GRK2 protein stability by blocking the ubiquitin–proteasome-mediated degradation.

### EIF3d exerts the tumor-promoting activities through GRK2-mediated activation of PI3K/AKT pathway

**Figure 6:**
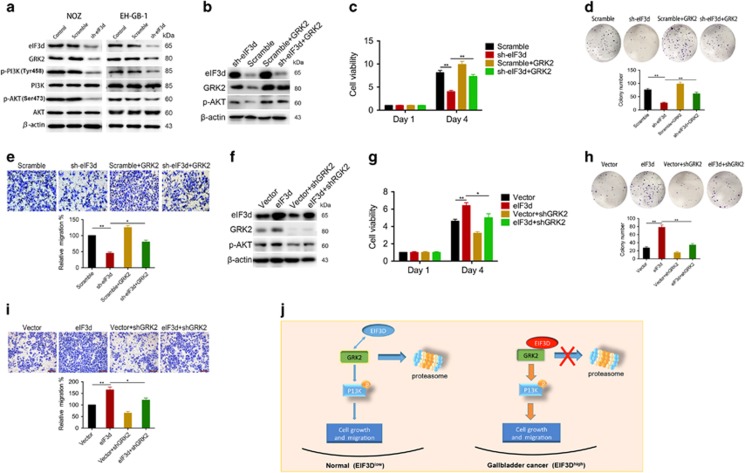
EIF3d exerts the tumor-promoting activities through the GRK2-mediated activation of PI3K/AKT signaling pathway. (**a**) The levels of phosphorylated PI3K, total PI3K, phosphorylated Akt, total Akt, eIF3d, and GRK2 were detected in control, scramble and sh-eIF3d cells by western blot analysis. *β*-Actin was used as the loading control. (**b**) ShRNA of eIF3d and GRK2 expression vector were used to knockdown eIF3d and reconstitutive express GRK2, respectively, in NOZ cells. The targeted protein levels were immunoblotted with specific antibodies as indicated. (**c**) Cell viability of NOZ cells expressing shRNA of eIF3d and shRNA of eIF3d plus GRK2 were determined by using CCK-8 assay. (**d**) Effects of eIF3d knockdown and reconstitutive expression of GRK2 in NOZ cells on colony formation capacity. Colony numbers were shown in the bar graph. (**e**) Transwell assay detected the migration ability in NOZ cells transfected with scramble, sh-eIF3d and sh-eIF3d+GRK2. (**f**) Western blot analysis of GRK2 and P-AKT in GBC-SD cells expressing eIF3d with or without treatment of GRK2 inhibitor methyl[(5-nitro-2-furyl)vinyl]-2-furoate at 2 *μ*M for 24 h. Cell viability (**g**) and colony formation ability (**h**) and cell migration ability (**i**) were determined in GBC-S.D. cells expressing eIF3d with or without treatment of GRK2 inhibitor. Error bars indicate the mean±S.E. of three independent experiments (*P<0.05, **P<0.01). (**j**) Proposed mechanistic scheme of eIF3d stabilize GRK2 to activate PI3K/AKT signaling pathway in GBC. Overexpression of eIF3d in GBC physically interacts with the GRK2 and, as a result, abrogates the ubiquitin–proteasome degradation of GRK2, which lead to activate PI3K/AKT signaling pathway, finally promote cell growth and migration

GRK2 is a ubiquitous member of the G protein-coupled receptor kinase (GRK) family that appears to have a central, integrative role in signal transduction cascades.^24^ In addition to participate in the regulation and signaling of a variety of G protein-coupled receptor (GPCR) activity, GRK2 has been shown to regulate a variety of proteins such as PI3K, MEK, ezrin or GIT. Consistently, splenocytes and T lymphocytes isolated from GRK2^+/−^ mice display increased agonist-induced activation of ERK and PI3K/Akt pathway.^25^ Stable overexpression of GRK2 in breast cancer cells significantly facilitated both mitogenic (ERK1/2) and pro-survival signaling (AKT) in response to heregulin or EGF.^26^ Our experimental data demonstrated that ablation of eIF3d can significantly decrease GRK2 protein level. Furthermore, compared with control cells, the levels of both phosphorylated PI3K and Akt were decreased in the eIF3d knockdown cells, whereas their total protein levels were unaffected (Figure 6a). Thus, we speculate that eIF3d regulates PI3K/AKT signaling pathway by control its upstream kinase GRK2 level. To explore the biological connection of GRK2 with the tumor-promoting function of eIF3d, we examined the effect of GRK2 re-expression in eIF3d knockdown stable NOZ cells on tumorigenicity. Reconstitutive expression of GRK2 can restore p-AKT expression level in the eIF3d knockdown NOZ cell (Figure 6b). Furthermore, restoration of GRK2 expression level significantly rescued the hyperproliferative phenotype, colony formation and migration capacities of the eIF3d knockdown NOZ cells, although it does not fully restored tumor malignancy as compared with the control group (Figures 6c and e). Consistent with these observations, treatment of the eIF3d-overexpressing GBC-SD cells with GRK2 inhibitor significantly abolished the upregulated p-AKT level induced by eIF3d (Figure 6f). Similarly, GRK2 inhibitor treatment attenuated the hyperproliferative status, colony forming and migration capacities in eIF3d-overexpressing GBC-SD cells (Figures 6g and i). Taken together, these data suggest that eIF3d promotes gallbladder tumor growth and migration at least partially through regulating GRK2 activity, which in turn leads to activation of PI3K/AKT signaling pathways (Figure 6j).

## Discussion

GBC is a highly invasive and rapidly proliferative cancer with a poor prognosis.^27^ Therefore, elucidating the molecular mechanisms underlying GBC progression is critical for the development of new therapeutic strategies for improving the outcome of patients with advanced GBC.

Herein, we for the first time demonstrate that eIF3d is involved in GBC, and this gene is highly expressed in GBCs as compared with the cholecystitis gallbladder epithelial tissues and gallbladder normal tissue adjacent tumor. The immunostaining intensity of eIF3d correlates positively with the tumor clinical stage and metastasis, and elevated expression of eIF3d significantly correlated with poor survival in GBC patients. Our results highly imply that eIF3d may be an important biomarker and thus RNA or protein quantification of eIF3d in gallbladder pathologic examination will predict prognosis of the GBC patients.

In this study, we revealed that eIF3d functions as an oncoprotein. Our findings demonstrated EIF3d promotes tumorigenesis by altering cyclin A and cyclin B1, which are key regulators for cell cycle, and by antagonizing apoptosis through regulating the caspase-dependent apoptosis pathways including bcl-2, caspase-3 and PARP. In addition to its tumorigenic roles, this study also showed that eIF3d enhances tumor metastasis *in vitro* and *in vivo*, indicating that overexpression of eIF3d may result in metastasis-related genetic alteration in GBC cell lines. Metastasis is a multistep cellular process and the most common cause of death in GBC patients. At the molecular level, the acquisition of genetic and/or epigenetic alterations, along with the cooperation of stromal cells, contribute to cancer metastasis.^28^ The underlying molecular mechanisms by which eIF3d promotes metastatic potential of GBCs may, at least part, involve EMT induced by elevated eIF3d in GBC. EMT is a crucial step in tumor progression and has a critical role during cancer invasion and metastasis. During this process, epithelial cells lose their properties and acquire mesenchymal phenotypes indicated by increased expression of mesenchymal-related markers, such as vimentin, and decreased expression of epithelial-related markers, such as E-cadherin.^29, 30, 31^ Knockdown of eIF3d enhances the E-cadherin expression and suppresses the N-cadherin and Vimentin expression. E-cadherin functions as an invasion suppressor, whereas N-cadherin and Vimentin promote cell motility and invasion in cancers.^32, 33^ Taken together, eIF3d promotes cell migration through modulating the key elements of EMT in GBCs.

Current study provides strong evidence for the oncogenic properties of eIF3d, including promotion of anchorage-independent growth, inhibition of apoptosis, increase of cellular proliferation and invasion *in vitro* and *in vivo*. Accordingly, we explored the underlying mechanisms of eIF3d in GBC oncogenesis, particularly the downstream signaling pathways. To define the regulatory scenario, we identify eIF3d-interacting proteins by using yeast two-hybrid screening and defined for the first time that GRK2 directly interacts with eIF3d.

GRK2 is a ubiquitous member of the GRK family that appears to have a central, integrative role in signal transduction cascades. GRKs participate together with arrestins in the regulation of GPCRs.^24^ GPCRs are founding members of the superfamily of seven transmembrane-spanning receptors that regulate physiologic and pathophysiologic processes, including initiation and progression of cancer. Overexpression and activating mutations of GPCRs are linked to tumor growth, angiogenesis and metastasis,^34, 35^ and targeting mutated or deregulated GPCRs are promising in experimental cancer therapy.^36, 37^ The GRKs constitute a group of protein kinases (seven members in mammals) that specifically recognize and phosphorylate agonist-activated GPCRs. GRKs are shown to be important regulator(s) specifically with respect to tumorigenesis and metastasis in many types of cancers including breast cancer, prostate cancer and pancreatic cancers.^38, 39^ Previous studies revealed that GRK2 protein levels can be either upregulated in tissue samples of patients with granulosa cell tumors and with differentiated thyroid carcinoma,^40^ or downregulated in a subgroup of prostate tumors.^41^ In addition, GRK2 has been recently shown to establish a complex network of novel functional interactions during cell cycle progression that are critical for timely G2/M transition.^42^ Ho *et al.*^43^ demonstrated that GRK2 phosphorylates Smad to inhibit activin/TGFb-mediated cell growth arrest and apoptosis in both normal and cancer liver cells. Moreover, GRK2 promotes changes in actin cytoskeleton and paxillin localization consistent with enhanced focal adhesion turnover and higher cell motility. Collectively, increased GRK2 expression facilitates migration toward fibronectin and GRK2 downregulation impairs migration.^44^

Regulation of GRK2 protein stability is an important mechanism for modulating its expression levels and activity. GRK2 is rapidly degraded by the proteasome pathway, and that GRK2 ubiquitination and turnover is enhanced by *β*_2_AR activation.^24^ Mdm2, an E3-ubiquitin ligase is involved in the control of cell growth and apoptosis, has a key role in GRK2 degradation.^45^ In this study, we revealed a novel non-translational factor function of eIF3d, which regulates GRK2 protein stability by blocking the ubiquitin–proteasome-mediated degradation. This finding suggests that eIF3d may promote GBC proliferation and migration by regulating the stability of GRK2. GRK2 is emerging as a key node in signal transduction pathways, displaying a very complex interactome. This kinase has been reported to associate with PI3K, clathrin, GIT, caveolin,^24^ MEK, RKIP,^46, 47^ ERK^25^ and MAPK.^48^ Our data further unveiled there is connection between eIF3d/GRK2 and PIK3/AKT signaling pathways in GBCs. Knockdown eIF3d decreased GRK2 expression, leading to reduction of phosphorylated PI3K and AKT. Restore expression of GRK2 can rescue p-AKT and malignant phenotype in eIF3d knockdown cells. These results suggest that eIF3d-GRK2 may be involved in the regulation of the PI3K/AKT signaling pathway. PI3K/Akt is a classical signaling pathway, and its activation induces cell growth,^49^ promotes EMT^50^ and stimulates Bax-mediated signaling for apoptosis progression.^51^ Our experimental results suggest that activation of PI3K/Akt signaling is a critically downstream event responsible for eIF3d–GRK2 signal axis in cell proliferation, migration and metastasis of GBCs.

In conclusion, our study uncovered for the first time the clinical and biological significance of eIF3d in GBCs. We also provide further evidence that eIF3d promotes the PI3K/AKT signaling pathway by enhancing the stability of GRK2 (Figure 6j). EIF3d may be used as a prognostic biomarker for predicting prognosis of GBC patients, and pharmacological targeting eIF3d-GRK2 signaling pathway may provide a potent treatment option for patients with GBCs.

## Materials and Methods

### Patients and clinicopathological data

Tumor tissue specimens were obtained from 92 GBC patients and 103 cholecystitis patients who had undergone radical cholecystectomy, without any prior radiotherapy or chemotherapy, between 2008 and 2014 at the Department of General Surgery, Xinhua Hospital, School of Medicine, Shanghai Jiao Tong University, Shanghai, China. Among the 92 gallbladder adenocarcinomas, the survival information for patients was collected through phone calls. In addition, 103 patients with cholelithiasis who underwent simple cholecystectomy were included as controls. The patients provided consent for the use of tumor tissue for clinical research and the Shanghai Jiao Tong University Xinhua Hospital Ethical Committee approved the research protocol. All diagnoses of GBC, cholelithiasis and lymph node metastasis were confirmed by histopathological examination, and all tissue samples were fixed in 4% formalin immediately after removal and were embedded in paraffin for immunohistochemical staining.

### Immunohistochemical analysis of GBC tissues

Following deparaffinization and quenching of endogenous peroxidase, sections were incubated with 1% bovine serum albumin (BSA) in PBS. Subsequently, the slides were treated with rabbit anti-human-eIF3d (1:200, Abcam, Burlingame, CA, USA) and mouse anti-human-GRK2 (1:100, ThermoFisher, Waltham, MA, USA) antibodies followed by incubation with goat anti-rabbit and goat anti-mouse IgG antibodies. The slides were counterstained with ChemMate Hematoxylin (DakoCytomation, Kyoto, Japan) and mounted and observed under a microscope (Leica, Wetzlar, Germany). Sections were semiquantitatively scored as described previously.^52^

### Cell lines, reagents, plasmids, transfection and lentivirus infection

Detailed descriptions of the cell lines, reagents, plasmids, transfection and lentivirus infection are available in the Supplementary Materials and Methods.

### Cell viability, cell cycle, cell apoptosis, clonogenic, wound-healing, transwell migration, immunoblotting, qRT-PCR

Detailed descriptions of the cell viability, cell cycle, cell apoptosis, clonogenic, wound-healing, transwell migration, immunoblotting and qRT-PCR assay are available in the Supplementary Materials and Methods.

### Immunofluorescence analysis

Transfected GBC cells were seeded onto glass slides for 48 h. The cells were then washed once with PBS and fixed with 4% paraformaldehyde in PBS for 20 min, permeabilized with 0.1% Triton X-100 in PBS for 30 min at room temperature followed by blocking with 3% BSA in PBS for 1 h. The cells then were stained with anti-eIF3d antibody (1:100 dilution, overnight at 4 °C). F-actin was stained with phalloidin (Beyotime Biotechnology, Shanghai, China). Cells were mounted with DAPI Fluoromount-G media with DAPI nuclear stain. Slides were viewed with confocal microscopy (Leica).

### Mouse xenograft and metastasis model

Female Balb/c nude mice (6–8 weeks of age, weighing 18–20 g) were purchased from the Shanghai Laboratory Animal Center of the Chinese Academy of Sciences (Shanghai, China). All mice were housed in specific pathogen-free (SPF) conditions following the guidelines of the Ethics Committee of Xinhua Hospital, School of Medicine, Shanghai Jiao Tong University. Before the experiment, mice were randomly divided into two groups (sh-eIF3d and Scramble). In all, 2 × 10^6^ NOZ cells were injected subcutaneously into the right flank of a nude mouse. After establishment of the nude mouse xenograft model, tumor sizes were measured every week using micrometer calipers. Tumor volumes were calculated using the following formula: tumor volume=width^2^ (mm^2^) × length (mm)/2, where width and length were the shortest and longest diameters, respectively. All nude mice were killed after 5 weeks from the first injection and the tumors were removed for IHC and western blot. To induce liver and lung metastasis, nude mice were injected with 1 × 10^6^ of EH-GB-1 cells (scramble and sh-eIF3d) by the spleen and lateral tail vein. After 1 month, the mice were killed and the lungs and livers were removed for HE staining.

### Yeast two-hybrid screening

Human eIF3d cDNA fragment was cloned into pGBKT7 vector and used to screen a pACT2-human fetal liver matchmaker cDNA library in a yeast two-hybrid system (Clontech, Mountain View, CA, USA). *β*-Galactosidase activities were measured using *o*-nitrophenyl-galactoside as a substrate. Then selected positive clones were validated by sequencing.

### Co-immunoprecipitation assay

Cells were washed with ice-cold phosphate-buffered saline and lysed in a lysis buffer (50 mmol/l Tris-HCl, pH 8.0; 150 mmol/l NaCl; 1% NP-40) supplemented with complete protein inhibitor cocktail (Roche, Penzberg, Germany). Cell lysates were incubated with 2 *μ*g rabbit anti-eIF3d antibody or mouse anti-GRK2 antibody overnight at 4 °C and precipitated with 20 *μ*l protein G-Sepharose 4 Fast flow (Thermo, Waltham, MA, USA) for 4 h at 4 °C. The co-immunoprecipitates were washed four times with the lysis buffer and boiled for 5 min at 100 °C in protein loading buffer. Immunoprecipitated proteins were detected by following immunoblots.

### Statistical analysis

The χ^2^ test or Fisher’s exact probability test was used to compare clinicopathological features of the patients with eIF3d expression. Kaplan–Meier plots and log-rank tests were used for survival analysis. Correlation analysis between eIF3d and GRK2 expression was evaluated using Spearman correlation analysis. Statistical analysis was performed with SPSS statistical software (SPSS Inc., Chicago, IL, USA). Data were reported as the means±S.E. when appropriate and *P*<0.05 was considered statistically significant.

## Figures and Tables

**Table 1:** Correlation of eIF3d expression with clinicopathological features in GBC specimens

**Characteristic**	**Patient # of EIF3D expression score**			***P-*****value**
	**Total**	**−/+**	**++/+++**	
*Age*				0.296
<64	44	12	32	
≥64	48	18	30	
				
*Gender*				0.675
Male	31	11	20	
Female	61	19	42	
				
*Tumor differentiation*				0.236
Well/moderate	35	14	21	
Poor	57	16	41	
				
*TNM stage (AJCC)*				**<0.001**
I/II	39	30	9	
III/IV	63	10	53	
				
*Liver metastasis*				**<0.001**
Present	57	9	48	
Absent	35	21	14	
				
*Lymph node metastasis*				**<0.001**
Present	50	10	40	
Absent	32	20	12	
				
*Microvascular and neural invasion*				0.1683
Present	56	17	39	
Absent	36	13	23	
Total	92	30	62	

Bold values indicate *P*<0.05.

**Table 2:** Correlation between eIF3d expression level and GRK2 expression level in GBC specimens (R=0.550, *P*<0.01)

	**eIF3d level**				
	**−**	**+**	**++**	**+++**	**Total**
*GRK2 level*					
−	3	2	1	0	6
+	3	13	6	2	24
++	0	5	16	7	28
+++	0	4	11	19	34
Total	6	24	34	28	92
