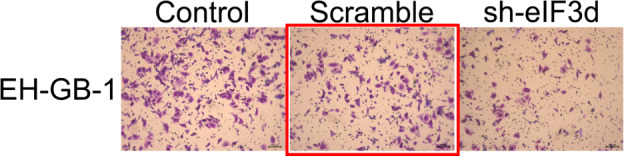# Correction: EIF3D promotes gallbladder cancer development by stabilizing GRK2 kinase and activating PI3K-AKT signaling pathway

**DOI:** 10.1038/s41419-021-04465-2

**Published:** 2021-12-20

**Authors:** Fei Zhang, Shanshan Xiang, Yang Cao, Maolan Li, Qiang Ma, Haibin Liang, Huaifeng Li, Yuanyuan Ye, Yijian Zhang, Lin Jiang, Yunping Hu, Jian Zhou, Xuefeng Wang, Yong Zhang, Lei Nie, Xiao Liang, Wei Gong, Yingbin Liu

**Affiliations:** 1 Department of General Surgery, Xinhua Hospital Affiliated to Shanghai Jiao Tong University School of Medicine, Shanghai, China; 2 Shanghai Research Center of Biliary Tract Disease, Shanghai, China; 3 Department of Molecular and Cellular Oncology, The University of Texas MD Anderson Cancer Center, Houston, TX USA; 4 Department of General Surgery, Sir Runrun Shaw Hospital Affiliated to Zhejiang University, Hangzhou, China

Correction to: *Cell Death & Disease* 10.1038/cddis.2017.263, published online 08 June 2017

Following the publication of this article, the authors noticed a mistake in Supplementary Figure 3A. Due to a mistake, the control group image for EH-GB-1 was accidentally used for the Scramble group image as well. All authors agree to the correction and apologize for this error. The correct Supplementary Figure 3A can be found below.

**Figure:**