# Supplementary material for: EIF3D promotes gallbladder cancer development by stabilizing GRK2 kinase and activating PI3K-AKT signaling pathway
Source: Cell Death Dis. 2017 Jun 8;8(6):e2868–. doi: 10.1038/cddis.2017.263 (PMC5520919; doi:10.1038/cddis.2017.263)
Supplement: Supplementary Information [file cddis2017263x1.docx]

**Supplementary Material and Methods**

**Cell culture and reagents**

The human GBC cell lines NOZ, EH-GB-1, EH-GB-2, SGC-996 and GBC-SD were purchased from the Cell Bank of Type Culture Collection of the Chinese Academy of Sciences (Shanghai, China). The OCUG-1 cell line was obtained from the Health Science Research Resources Bank (Osaka, Japan). NOZ cell line were maintained in William’s medium (Gibco, Grand Island, NY, USA) supplemented with 10% fetal bovine serum (FBS; Gibco). GBC-SD cells were maintained in DMEM medium (Gibco) containing 10% FBS. EH-GB-1, EH-GB-2, SGC-996 and OCUG-1 cells were cultured in RPMI 1640 (Gibco) supplemented with 10% FBS. These cell lines were cultured at 37°C in a humidified incubator containing 5% CO_2_. Proteasome inhibitor MG-132 and CHX (Cycloheximide from microbial) were purchased from Sigma (Louis, MO). GRK2 inhibitor methyl[(5-nitro-2-furyl)vinyl]-2-furoate was purchased from Calbiochem (La Jolla, CA)

**EIF3D knockdown and overexpression**

Oligonucleotides were synthesized to generate annealing shRNA targeting human eIF3d (NM_003753.3)

(5′-GCGTCATTGACATCTGCATGACTCGAGTCATGCAGATGTCAATGACGCTTTTTT-3′) and then the fragment was subcloned into pFH1UGW vector (Addgene). Lentiviral stocks were prepared by co-transfecting HEK-293T cells with eIF3d shRNA and standard virus packaging systems. GBC cells were infected with filtered lentivirus plus 4 μg /mL polybrene (Sigma-Aldrich). EIF3d cDNA were amplified by PCR from cDNA of gallbladder cancer cell NOZ. Constructs encoding eIF3d and eIF3d truncation were subcloned into pcDNA3 vector (Addgene). Overexpression vector was transfected into cells by lip2000 (Invitrogen).

**Cell viability, cell cycle and cell apoptosis**

Cell viability was determined at 0, 1, 2, 3, 4, 5, 6 day using the Cell Counting Kit-8 reagent (Dojindo) according to the manufacturer’s protocol. Fluorescence-activated cell-sorting (FACS) for cell cycle and apoptosis was performed using propidium iodine (Invitrogen) and human Annexin V-FITC Kit (Invitrogen), respectively, according to the manufacturer’s protocol.

**Wound healing and cell migration assay**

GBC cells in medium containing 10% FBS were seeded into wells of 6-multiwell plates (Becton Dickinson). After the cells grew to 100% confluence, the wound was made by scraping a conventional pipette tip across the monolayer. Cells were washed with PBS and refreshed with medium with or without 10% FBS. After 12h incubation at 37°C, the cells were fixed and photographed. Cell migration assay were performed using Boyden chamber (BD Biosciences). 4×10^4^ Cells were seeded at the upper chamber in DMEM without FBS. In the lower chamber, 600ul of DMEM with 10% FBS were added. After 12h of incubation, cell were fixed and stained with crystal violet. Cells in the upper chamber were removed and cells migrating through the membrane were photographed in three randomly selected fields and counted using ImageJ software (NIH).

**Two dimensional and three-dimensional Colony formation assay**

Colonogenic survival assays were performed by plating approximately 200-400 cells in 6-well culture dishes for 8-12 days. Cells were then fixed with 4% paraformaldehyde, stained with crystal violet solution and formed colonies (≥50 cells) were visually counted.

Anchorage- independent growth was assessed by colony formation ability in soft agar. 500 cells were suspended in soft agar mixture (William’s medium, 10% fetal bovine serum, and 0.35% agar) and subsequently were overlaid on the solidified 0.5% agar base in six-well plates. After 2-3 weeks, colonies were observed under the microscope in 10 fields per well. Triplicate independent experiments were performed.

**Real-time quantitative PCR (qRT-PCR)**

Total RNA was extracted using the Trizol LS reagent (Invitrogen). The mRNA was reverse-transcribed using the SuperScript First-Strand Synthesis System (Invitrogen). PCR amplification was performed using the following primers: eIF3D, 5’-CTGGAGGAGGGCAAATACCT-3’ (sense) and 5’-CTCGGTGGAAGGACAAACTC-3’ (antisense); and β-actin, 5’-TTAGTTGCGTTACACCCTTTC-3’ (sense) and 5’-ACCTTCACCGTTCCAGTTT3’ (antisense). Real-time quantitative RT-PCR was performed following a standard SYBR Green PCR protocol using SYBR® Premix Ex Taq™ (Takara, Dalian, China). The relative eIF3d mRNA level was normalized to housekeeping gene β-actin.

**Immunoblotting**

Three days after infection by lentivirus or transfected by lip2000, whole cell lysates were prepared and the proteins were separated by SDS-PAGE and then transferred to polyvinylidene diﬂuoride (PVDF) membranes (Millipore, Bedford, MA, USA). The membranes were blocked and then probed with specific antibody against eIF3d (1:2000 dilution, Abcam, Cambridge, UK), GRK2 (1:1000 dilution, ThermoFisher) and β-actin (1:5000 dilution, Santa Cruz Biotechnology, Santa Cruz, CA, USA). After washing, the membranes were incubated with horseradish peroxidase-conjugated goat anti-Rabbit IgG or goat anti-mouse IgG (Santa Cruz) and visualized using the enhanced chemiluminescent (ECL) detection reagent from Pierce (Rockford, IL, USA).
