# Supplementary figures and images for: EIF3D promotes gallbladder cancer development by stabilizing GRK2 kinase and activating PI3K-AKT signaling pathway
Source: Cell Death Dis. 2017 Jun 8;8(6):e2868–. doi: 10.1038/cddis.2017.263 (PMC5520919; doi:10.1038/cddis.2017.263)

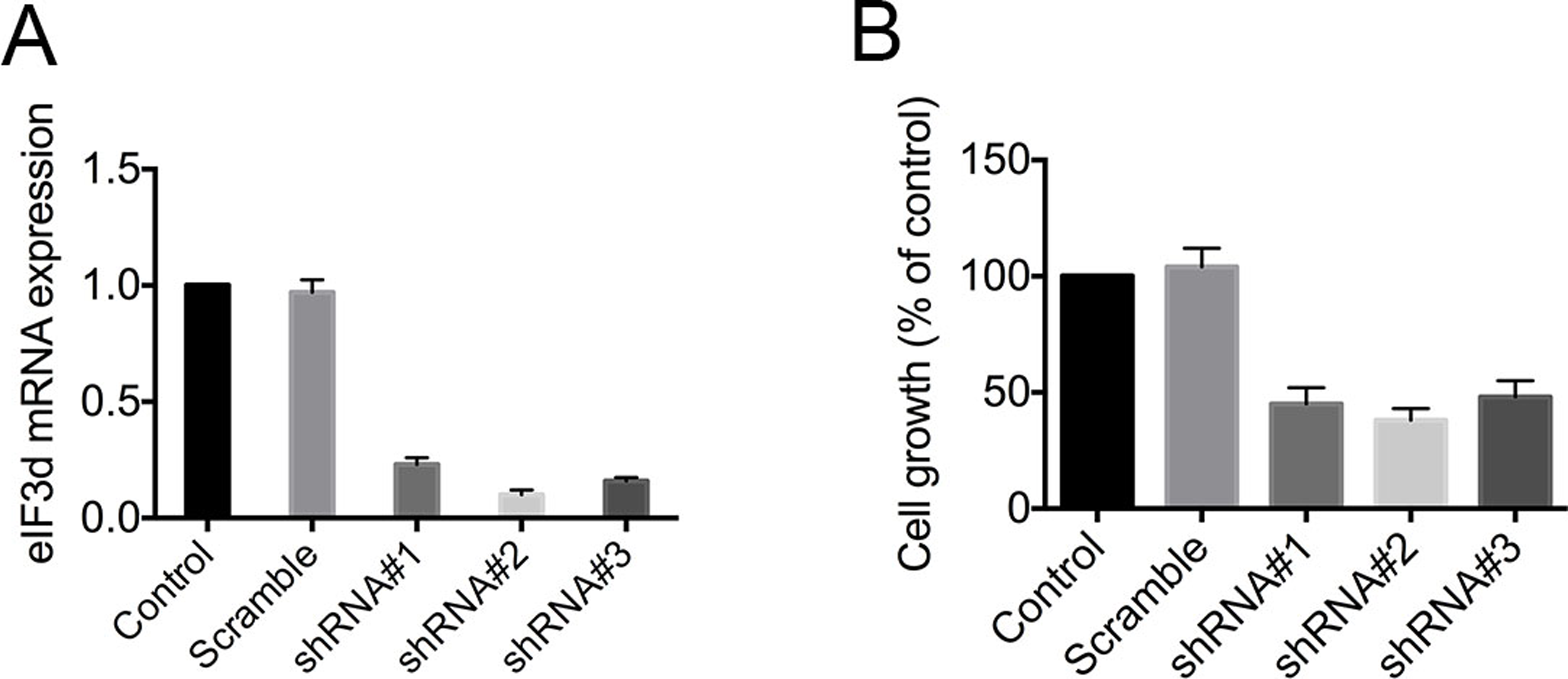

Supplement: Supplementary Figure 1 [file cddis2017263x2.tif]

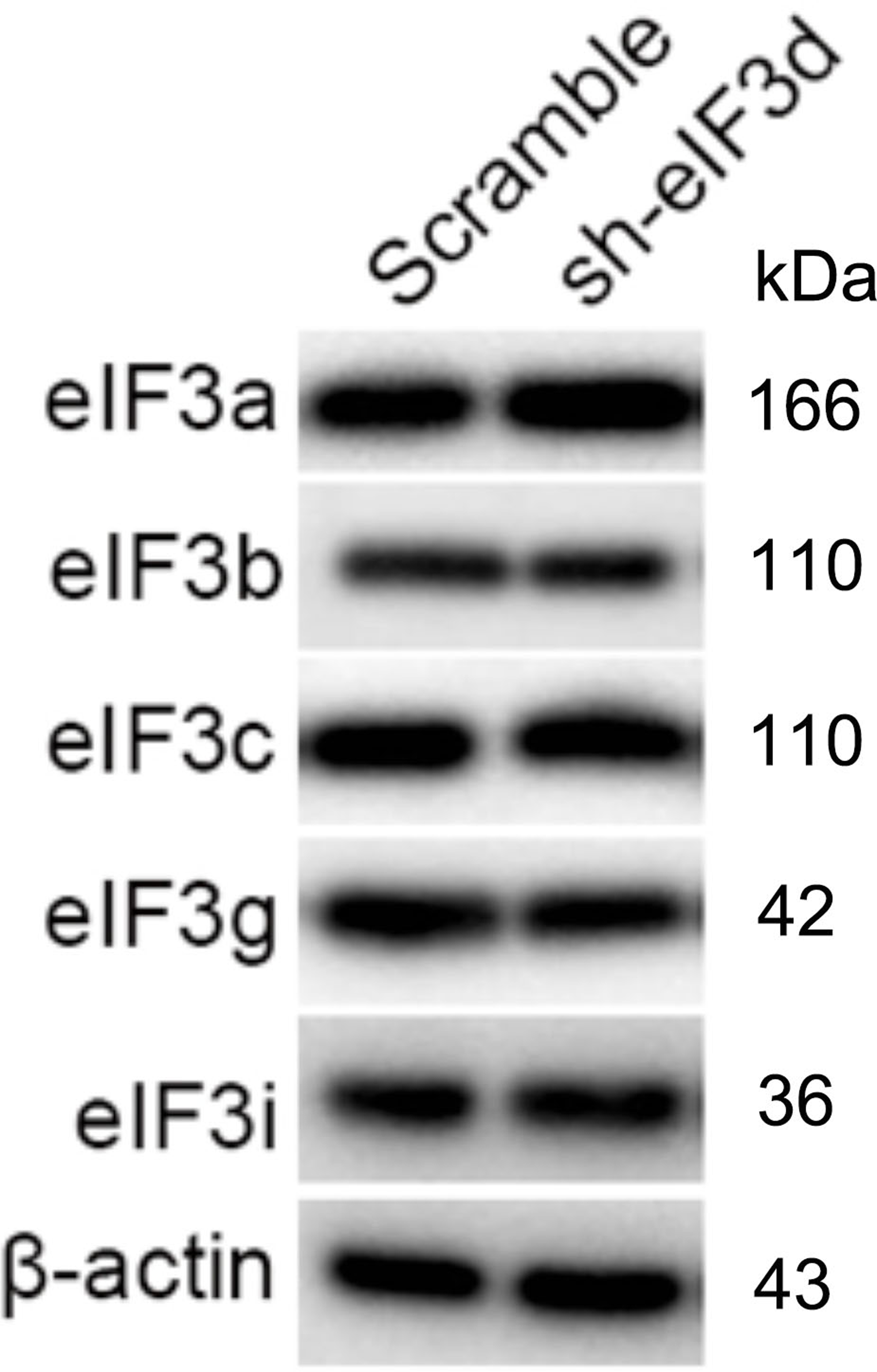

Supplement: Supplementary Figure 2 [file cddis2017263x3.tif]

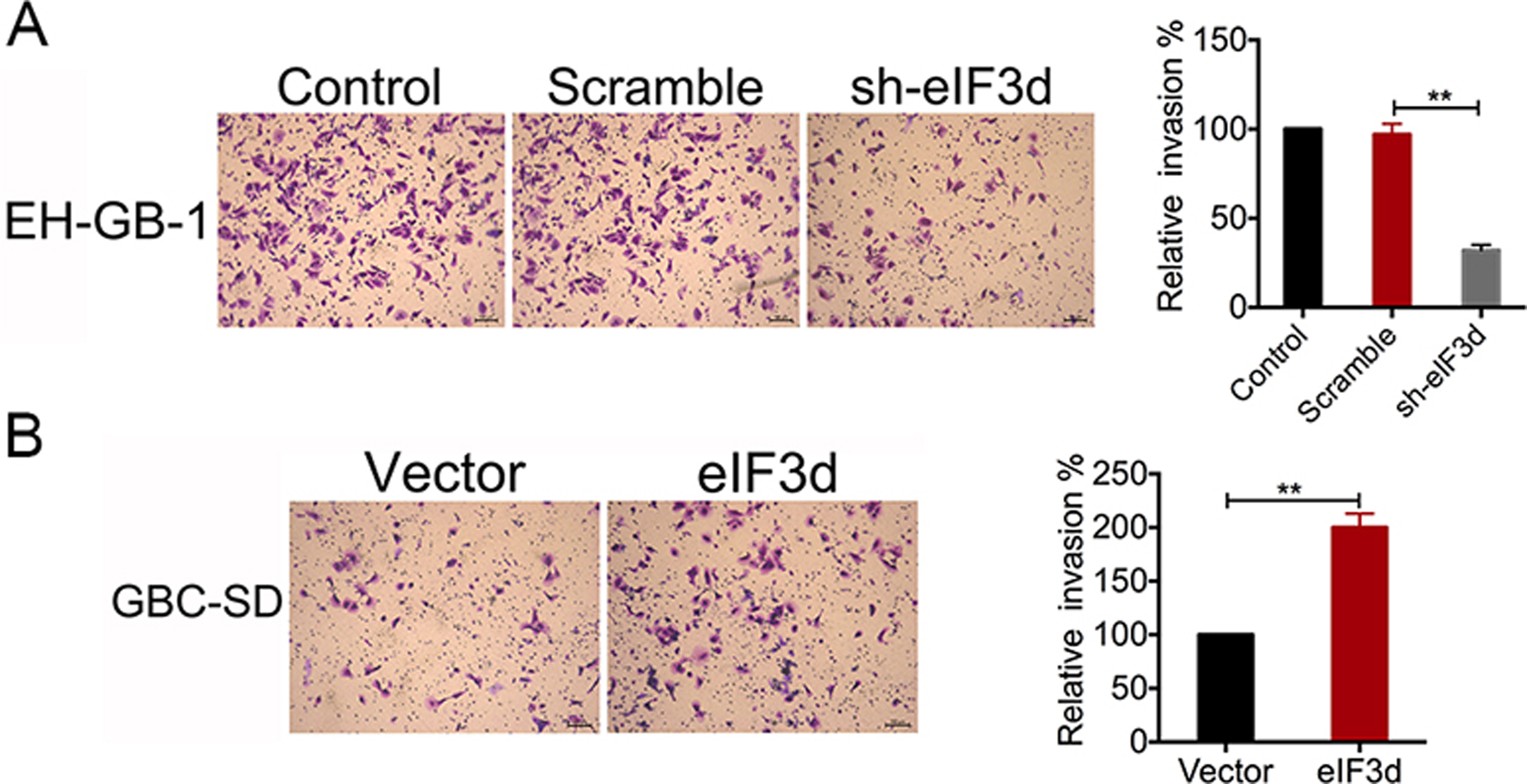

Supplement: Supplementary Figure 3 [file cddis2017263x4.tif]

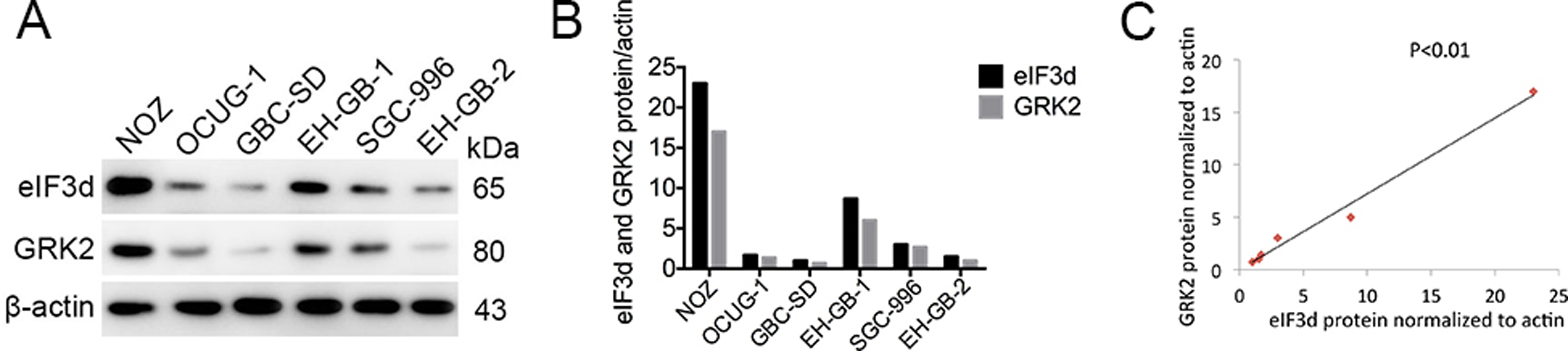

Supplement: Supplementary Figure 4 [file cddis2017263x5.tif]
